# Revival of Leishmanization and Leishmanin

**DOI:** 10.3389/fcimb.2021.639801

**Published:** 2021-03-17

**Authors:** Thalia Pacheco-Fernandez, Greta Volpedo, Sreenivas Gannavaram, Parna Bhattacharya, Ranadhir Dey, Abhay Satoskar, Greg Matlashewski, Hira L. Nakhasi

**Affiliations:** ^1^ Departments of Pathology and Microbiology, Wexner Medical Center, The Ohio State University, Columbus, OH, United States; ^2^ Division of Emerging and Transfusion Transmitted Diseases, Center for Biologics Evaluation and Research (CBER), Food and Drug Administration (FDA), Silver Spring, MD, United States; ^3^ Department of Microbiology and Immunology, McGill University, Montreal, QC, Canada

**Keywords:** leishmanization, leishmanin, vaccine, immunity, leishmaniasis

## Abstract

Leishmaniasis includes a spectrum of diseases ranging from debilitating cutaneous to fatal visceral infections. This disease is caused by the parasitic protozoa of the genus *Leishmania* that is transmitted by infected sandflies. Over 1 billion people are at risk of leishmaniasis with an annual incidence of over 2 million cases throughout tropical and subtropical regions in close to 100 countries. Leishmaniasis is the only human parasitic disease where vaccination has been successful through a procedure known as leishmanization that has been widely used for decades in the Middle East. Leishmanization involved intradermal inoculation of live *Leishmania major* parasites resulting in a skin lesion that following natural healing provided protective immunity to re-infection. Leishmanization is however no longer practiced due to safety and ethical concerns that the lesions at the site of inoculation that can last for months in some people. New genome editing technologies involving CRISPR has now made it possible to engineer safer attenuated strains of *Leishmania*, which induce protective immunity making way for a second generation leishmanization that can enter into human trials. A major consideration will be how the test the efficacy of a vaccine in the midst of the visceral leishmaniasis elimination program. One solution will be to use the leishmanin skin test (LST) that was also used for decades to determine exposure and immunity to Leishmania. The LST involves injection of antigen from *Leishmania* in the skin dermis resulting in a delayed type hypersensitivity (DTH) immune reaction associated with a Th1 immune response and protection against visceral leishmaniasis. Reintroduction of novel approaches for leishmanization and the leishmanin skin test can play a major role in eliminating leishmaniasis.

## Highlights

Newer technologies have made the live leishmanization vaccine and the leishmanin skin test safer and re-introduction of these interventions can support the elimination of leishmaniasis.

## Introduction

Leishmaniasis includes a spectrum of diseases ranging from disfiguring cutaneous to fatal visceral infections. This disease is caused by the parasitic protozoa of the genus *Leishmania* that is transmitted by infected sandflies. Over 1 billion people are at risk of leishmaniasis with an annual incidence of over 2 million cases throughout tropical and subtropical regions in close to 100 countries. Strategies to eliminate Visceral Leishmaniasis in the Indian subcontinent, that has a current goal of reducing the incidence of VL to below 1/10,000 of population by the year 2020, is centered on rapid detection and treatment of VL to reduce the number of human reservoirs, and vector control using indoor residual spraying (Sundar et al., 2018). Such elimination programs in endemic areas have yet to achieve lasting impact, and the need for appropriate diagnostic, treatment and prevention methods against VL to ensure long term sustainability and prevent reemergence of VL is recognized (Sundar et al., 2018). Extensive studies characterizing the immune response in resistant and outbred experimental animal models have illuminated the immune mechanisms of protection in cutaneous and visceral leishmaniasis (Kaye and Scott, 2011). Yet, very few vaccines against leishmaniasis have reached clinical trials (Moafi et al., 2019). Leishmaniasis is the only human parasitic disease where vaccination has been successful through a procedure known as leishmanization that has been widely used for decades in the Middle East (Khamesipour et al., 2005). Thus there is an increased recognition that a safer Leishmanization strategies may yield efficacious vaccines and help achieve elimination targets. A brief but not exhaustive review of the immune responses reported in experimental animal models and human studies and the vaccination strategies pursued so far against Leishamaniasis are discussed in the following sections. A discussion on the utility of Leishmanin skin test (LST) as a surrogate for measuring vaccine response in the future clinical trials is also included.

## Immune Responses in Leishmaniasis

Leishmaniasis is a parasitic disease that affects more than 12 million people in the word and is caused by the intracellular protozoa of the genus *Leishmania* (Centers for Disease Control and Prevention (CDC), 2020). There are over 20 species of *Leishmania* parasites which are transmitted to the host by the bite of female phlebotomine sandflies (World Health Organization (WHO), 2020). The main clinical manifestations of leishmaniasis are the cutaneous leishmaniasis (CL), and visceral leishmaniasis (VL) (Centers for Disease Control and Prevention (CDC), 2020). Generation of an effective immune response during both CL and VL requires the coordinated action of numerous cell types. A critical first step is the activation of cells of the innate immune system, including neutrophils, macrophages and dendritic cells. Neutrophils are the first responders against *Leishmania*, but they are also the first reservoir for the parasite before they reach their final host, the macrophages. Macrophages are the major host cell targeted by *Leishmania* parasites, which survive and replicate within these cells by manipulating their antimicrobial effector activity. The clearance of parasites by macrophages depends on activation of an appropriate immune response, which is usually initiated by dendritic cells (DCs). Recent evidence also suggests that innate cells modulate the adaptive immune response through the release of chemokines and cytokines necessary to activate T cells.

## Cutaneous Leishmaniasis

Cutaneous leishmaniasis (CL) is caused mainly by the parasites *L. major* and *L. tropica* in the Old World and *L. amazonensis*, *L. mexicana*, *and L. brazilensis* in the New World. These parasites cause painless skin ulcers that can be self resolve [localized cutaneous leishmaniasis (LCL)] or turn into chronic lesions present in different parts of the body [diffuse cutaneous leishmaniasis (DCL)], depending on the causative species and immune response (Scott and Novais, 2016). For the purposes of brevity, this review focuses solely on Old World *Leishmania* species. The immunopathogenesis of New World leishmaniasis, caused by infection with parasites such as *L. braziliensis* and *L. amazoniensis* is distinct from that caused by *L. major* in significant ways including sensitivity to IFN-β and TNF (Khouri et al., 2009; Novais et al., 2009). The immunological characteristics between the different CL causing species are out of the scope of this review.

A protective immune response against CL is characterized by activation of antigen presenting cells (APCs), such as DCs, and subsequent induction of T helper 1 (Th1)-polarized responses associated with IFN-γ production (Martínez-López et al., 2018). Innate immune cells that are recruited to the dermis following a sand fly bite also contribute to innate immunity. Clinical studies have found that low levels of macrophage chemotactic factor (MCP-1/CCL2) have been associated with DCL, while high levels have been found in LCL patients (Ritter et al., 1996). A previous study has shown that *L. major* promastigotes induce maturation in human dendritic cells (DCs), resulting in the increase of MHC-II and co-stimulatory molecules. These mature DCs also display increased production of IL-12p70 in a CD40L-dependent manner, which in turn can elicit a Th1 response characterized by interferon (IFN)-γ in autologous T cells derived from sensitized individuals (Marovich et al., 2000).

While a Th1 immune response is protective in murine models of CL, a Th2-polarized response, characterized mainly by IL-10, IL-4, and IL-13, confers susceptibility (Martínez-López et al., 2018). Although this dichotomy has been well documented throughout the years, there is a growing body of evidence suggesting that this paradigm might not be as clear-cut (Kaye and Scott, 2011). One study found that T-regs from the lesions of CL patients produce IL-10 and TGF-β. These cytokines reduce Th1 and macrophage activity, fostering a permissive environment for the growth of *Leishmania* parasites. Tregs also suppress proliferation and IFN-γ production of PBMCs-derived allogeneic CD4+ T cells *in vitro* (Mougneau et al., 2011; Gupta et al., 2013). Lastly, Th17 cells can play a role in balancing the pro and anti-inflammatory responses in experimental models as well as in patients (Gonçalves-de-Albuquerque et al., 2017). The recruitment of the adaptive immune cells to the site of infection by the coordinated action of various APCs that secrete different chemokines in healed CL lesions (Geiger et al., 2010). Similarly, a high expression of CXCR3, a chemokine receptor involved in the recruitment of Th1 cells, was also found in the early stages of CL localized lesion in human patients (Campanelli et al., 2010). A preclinical study showed that multi-functional Th1 cells thus recruited produce IFN-γ, IL-2, and TNF-α, shown to mediate protection as suggested in pre-clinical studies (Darrah et al., 2007).

In addition to cytokines, other factors such as Micro RNAs (miRs) that have been shown to regulate the expression of several immunologically relevant gene products, affect the immune response against CL. miRs are small non coding RNA molecules that play a role in silencing and post transcriptional regulation. Higher levels of miR-7, miR-146b, miR-133a, miR-223, and miR-328, predicted to regulate inflammasome genes, were found in the plasma of CL patients compared to healthy controls (Mendonça et al., 2020). miR-182 and miR-10a on the other hand, have been shown to regulate Th1- or Th2-associated Treg cells, respectively, and modulate their stability and suppressor functions in an *L. major* murine model (Kelada et al., 2013). Lemaire et al. (2013) reported an extensive analysis of the expression profiles of 365 miRs in human primary macrophages infected with *L. major*. They identified 64 miRs involved in macrophage fate during infection.While the earlier studies focused on the miRs as biomarkers of various disease states, understanding and modulating the different miRs involved can aid the development of new treatments as was shown by the therapeutic interventions targeting miR-21 in such conditions as cardiac hypertrophy, SLE, and psoriasis (Sheedy, 2015). Simiarly, miRs may hold potential as therapeutic targets against all form of Leishmaniasis including CL.

Studies with healed CL mouse models revealed that during the first stage of CL infection, both protective CD4+ T effector (T_EFF_) and CD4+ T central memory (T_CM_) cell pools are generated concurrently (Colpitts and Scott, 2010). Immunization with non‐persistent DHFR-TS null mutant parasites has been shown to protect against *Leishmania* infection in suceptible mouse models where the protection was mediated by T_CM_ poulations. T_CM_ populations persist after the antigen is cleared and are sequestered in the lymph nodes where they can differentiate into effector cells and proliferate upon re-infection (Zaph et al., 2004; Glennie and Scott, 2016). Effector memory T cells (T_EMs_) require parasitic persistence and migrate to peripheral sites where they can produce both Th1 and Th2 cytokines. Studies in susceptible murine models such as Balb/C showed that immunization with avirulent and non-persistent phosphomannomutase‐deficient *L. major* parasites induced protection that was mainly mediated by suppression of IL-10 and IL-13 but without significant difference in CD44+ CD62L^hi^ memory precursor populations compared to non-immunized controls (Kedzierski et al., 2008). A combination of T_CM_ and T_EM_ have been identified in patients who have healed from cutaneous lesions, suggesting a role for these two T cell subsets in long term protective immunity (Valian et al., 2013). Similarly, patients with active cutaneous lesions showed high levels of CD4+ (T_EM_) and CD8+ (T_EMRA,_ CD45RA^+^ T effector memory cells) effector memory T cells; in particular, the numbers of T_EMRA_ were higher in individuals with active disease compared to the healed and asymptomatic group, although T_EMRA_ from cured patients displayed a more robust response. Cured patients also had increased levels of IFN-γ-producing Th1 cells showing cytotoxic properties (Egui et al., 2018). A comprehensive understanding of the subpopulations of memory T cells at play in human leishmaniasis is crucial for the design of an effective vaccine. A non-live vaccine, for instance, will not be able to persist in the host, leading to the production of T_CM_ but not T_EM_ (Gollob et al., 2005). It has been shown that chronic parasite infection maintains Ly6C+CD4+effector T cells, and upon challenge with wild type *L. major* parasites, these are essential for IFN-γ production that mediates protection (Peters et al., 2014). Our studies in CL showed that upon challenge with wild type parasites, mice immunized with *LmCen* ^−/−^ parasites produced similar percentages of CD4+Ly6C+IFN-γ+ effector T cells to the healed mice (Zhang et al., 2020).

Healed CL models revealed that in addition to T_CM_ and T_EM_, tissue resident memory T cells (T_RM_) play crucial role in protection against leishmaniasis (Carvalho et al., 2013). T_RM_ are generated during the early stages of infection and can migrate pervasively through the skin, where they produce protective cytokines such as IFN-γ and recruit T_EFF_ upon re-stimulation. Due to the isotropic distribution of T_RM_ populations in skin and their capacity to respond quickly, T_RM_ populations are of great interest as mediators of protection. Unlike T_CM_ populations that are sequestered in secondary lymphoid organs following lesion resolution and thus require additional chemokine and cytokines cues to arrive at the site of re-infection after chemokine and cytokines gradients are produced at the infection site by the activities of tissue resident macrophages and neutrophils patrolling the organs, T_RM_ populations can swing into action more readily due to their indefinite presence in the skin likely at the site of reinfection. Due to the kinetics of recruitment and activity of T_RM_ populations precede that of T_CM_ populations, the former are investigated with great interest (Glennie et al., 2017; Ismail et al., 2020). Both T_CM_ and T_RM_ have been shown to transfer immunity to naïve animals (Glennie et al., 2015; Mackay and Carbone, 2015; Glennie and Scott, 2016). However, several pre-clinical studies show that this immunity can be partially or completely lost once the primary infection is cleared (Peters et al., 2014). In a murine leishmanization model, a subset of IFN-γ producing CD4+ T cells gathered at the site of sand fly challenge was shown to provide protection against *L. major* re-infection (Peters et al., 2009). More recently, studies showed that a short-lived IFN-γ producing T_EFF_ pool that are not derived from memory T cells confer significant protection and can be used as a protection biomarker (Peters et al., 2014; Hohman and Peters, 2019). These observations highlight that IFN-γ producing CD4+ T cells generated from T_CM_, T_RM_ or T_EM_ all play critical roles in mediating protective immunity against re-infection and that these populations need to be maintained in the host to induce protection.

## Visceral Leishmaniasis

The visceral manifestation of leishmaniasis, also called kala-azar, is caused by the *Leishmania* species *L. donovani* and *L. infantum/L. chagasi* (Centers for Disease Control and Prevention (CDC), 2020). Even though most cases of *leishmania* infections are sub-clinical, or even asymptomatic, there is a high percentage of fatality (95%) in untreated patients who develop VL (Chakravarty et al., 2019; World Health Organization (WHO), 2020). If it is not treated, the infection can extend to lymph nodes, spleen and liver, leading to the clinical manifestations of VL: hepatomegaly, splenomegaly, fever, weight loss, fatigue, anorexia, anemia, and finally, death (Saha et al., 1991; Soong et al., 2012; Kumar et al., 2019, Centers for Disease Control and Prevention (CDC), 2020).

Similar to CL, a Th1 polarization over a Th2 immune response and, most recently recognized, the balance of the Th17 response, are key factors for the development of resistance against VL (Dayakar et al., 2019). IFN-γ production by T cells is elicited by APC-derived IL-12, described above in the CL section (Ghalib et al., 1995; Nylén and Sacks, 2007). *In vitro* antigen stimulation of PMBCs from subjects healed from *L. chagasi* infection causes an increase of IL-12 concentration and elicits lympho-proliferation (Bacellar et al., 2000). Similarly, culture supernatants of PBMCs of from active VL patients in VL endemic regions showed significantly higher stimulation of IFN-γ in response to *L. donovani* (Ld1S) parasite antigen stimulation (Avishek et al., 2016). Moreover, IFN-γ production seems to be a determining factor for severity of the disease, as asymptomatic patients show higher numbers of IFN-γ producing cells compared to those patients who develop VL (Hailu et al., 2005). In fact, low levels of IFN-γ lead to the development of VL in patients with subclinical infection (Soong et al., 2012).

Together with the Th1 immune response, recent data suggest an important role for the Th17 immune response. A study of the Sudanese population following a VL outbreak, showed that the re-exposure of PBMCs to *L. donovani* antigen caused the production of IL-1β, IL-23, and IL-6; leading to the increase of IL-17 and IL-22 and the maintenance of Th17 cells. Interestingly, increased IL-17 production in these subjects correlates with protection and resistance against VL. (Pitta et al., 2009). A murine *L. donovani* infection model shows that IL-17 supports parasite clearance by enhancing IFN-γ and NO production; suggesting that both Th17 and Th1-mediated responses are necessary for the protection against VL (Ghosh K., et al., 2013). In contrast, another study reported that IL-17A-/- mice infected with *L. donovani* showed an increase in IFN-γ production by CD4+ T cells and better resistance against infection, suggesting that IL-17 promotes susceptibility to *L. donovani* infection. These mice also showed a reduced accumulation of neutrophils in the spleen and liver in the chronic phase of infection, and a decreased production of IL-4 and granuloma formation in spleen (Terrazas et al., 2016). In contrast, IL-17 was shown to contribute to the development of Th1 mediated protective imunity following immunization. Neutralization of IL-17 abrogated protective immunity indicating that IL-17 may have dual roles in pathogenesis and protection (Banerjee et al., 2018). The paradoxical roles of IL-17 and interaction between neutrophils, Th17, and Th1 response in VL and CL are reviewed elsewhere (Gonçalves-de-Albuquerque et al., 2017).

Distinct from CL, a mixed Th1/Th2 response has been reported in multiple human VL studies. For example, IL-27 is increased in the plasma of VL patients, and it is necessary for the development of IL-10 producing T cells (Ansari et al., 2011). It has been observed that IL-12-dependent IFN-γ production is inhibited by the addition of IL-10 into PMBCs cultures from the patients; while TGF-β does not seem to have a direct effect on IFN-γ inhibition (Bacellar et al., 2000; Caldas et al., 2005). Furthermore, IL-10 can make macrophages unresponsive to activation signals and decreases their TNF-α and NO production, allowing for amastigote replication (Nylén and Sacks, 2007). Recent data suggest that early increase in IL-10 inhibits host anti-*Leishmania* response; and the decrease of the IFN-γ/IL-10 ratio is associated with VL susceptibility (Mesquita et al., 2018). These data are supported by several studies in VL patients, where high levels of IL-10 were found in the plasma, sera, and lesion tissue from patients with active VL and correlate with parasitic load (Caldas et al., 2005; Hailu et al., 2005; Ansari et al., 2006; Kurkjian et al., 2006; Nylén et al., 2007; Verma et al., 2010). It is important to point out that IL-10 production by CD4+ T cells and DCs prevents the disruption of splenic architecture, even when it is associated with a poorer control of parasitic growth (Bunn et al., 2018).

Other Th2 anti-inflammatory cytokines, such as IL-4 and IL-13 have been shown to have a protective role in VL immunity, which is not related to the inhibition of IFN-γ (Satoskar et al., 1995; Murray et al., 2005). Mouse and hamster VL models infected with *L. donovani* have shown an increased expression of IL-4 (Moulik et al., 2020), which does not correlate with an increase of parasitic burden (Alexander et al., 2000). In humans, IL-4 has been related to patients with pre-clinical or asymptomatic infections; whereas it is rarely detected in patients with VL (Babaloo et al., 2001; Hailu et al., 2005). It is been demonstrated that, together with IL-13, IL-4 modulates the formation of mature granulomas in the liver by regulating collagen disposition (Stäger et al., 2003). Both, IL-13 and IL-4 thus are necessary for the efficacy of anti-leishmania chemotherapy (Alexander et al., 2000; McFarlane et al., 2011).

The Th1/Th2 balance is also regulated by miRs, and the expression profile is different between the *leishmania* strains *L. major and L. donovani* (Geraci et al., 2015). In the case of VL, miR155 favors the development of the Th1 response and IFN-γ production by targeting Th1 suppressors. Another study identified the miRs miR-29-b, miR-29a as suppressors of Th1 response. It has been shown that miR-135, miR-1272 and miR-155 act as suppressors of the response to IL-4 and IL-13 (Pandey et al., 2016). *L. donovani* infection has been shown to alter miR-122 which influences the cholesterol biosynthetic pathways in the host, that was previously shown to be critical for controlling the liver and splenic parasite burdens (Ghosh J., 2013). Other studies have analyzed large number of miRs to identify the changes in pathways related to T cell polarization. MiRs profile differences have been identified between post-kala-azar dermal leishmaniasis and VL (Kumar et al., 2020); as well as between *L. major* and *L. donovani* models (Geraci et al., 2015). In *L. donovani* infection, macrophages increase the expression of miRs such as mir-3620, mir-6385, mir-6973a, mir-6996, mir-328, mir-763, mir-6540, mir-1264, mir-3473f, and mir-8113 that permit parasite survival by downregulating the immune effector functions of host macrophages (Tiwari et al., 2017). Studies in murine and *ex vivo* human infections of DCs and macrophages with *L. donovani* showed that miR-21 expression was sigificantly induced upon infection and attenuates IL-12 mediated induction of Th1 immunity. Further, exosomes released from such infected cells similarly attenuated IL-12 expression and CD4 T cell proliferation indicating the role of miR-21 in shaping early immunity (Gannavaram et al., 2019). The identification of the miRs responsible for the regulation of the immune response has made it possible to design therapies that target specific signaling pathways to avoid setting a permissive environment during VL (Pandey et al., 2016).

## Vaccination Strategies Against Leishmaniasis

The immunological mechanisms of *Leishmania* pathogenesis as outlined in the previous sections have guided the development of several experimental vaccines against leishmaniasis. The vaccination strategies explored against leishmaniasis ranged from recombinant antigens, DNA vaccines, salivary gland proteins, killed parasites, and live attenuated parasites. The rich history of anti-leishmanial vaccines has been covered extensively in previous review articles on this theme (Kedzierski, 2011; Iborra et al., 2018; Kaye et al., 2020; Zabala-Peñafiel et al., 2020). For the purpose of brevity, a discussion on all the experimental vaccines is not being attempted in this article. Of all types of anti-leishmanial vaccines, only a handful of the candidate vaccines for dogs and humans entered clinical trials. The characteristics of these vaccines such as Leishvaccine, ALM, Leishmune, Canileish, GALM, LEISH-F1, LEISH-F2, LESH-F3, Leish-Tec, SMT+NH, and ChAd63-KH have been reviewed recently (Moafi et al., 2019). The choice of antigen and the adjuvant have been shown to greatly impact the vaccine response (Duthie and Reed, 2017). Studies with pre-clinical experimental models indicated that the recombinant antigen vaccines such as LEISH-F3+GLA-SE adjuvant could elicit IFN-γ, TNF-α, and IL-2 response in Phase-I studies in humans (Coler et al., 2015). The most recent iteration of this series of recombinant antigen vaccines is a variant of LEISH-F3 to which a third antigen derived from cysteine protease (CPB) was added based on the observation that this antigen was recognized by individuals with latent *Leshmania* infection (Duthie et al., 2017). The ability of viral vectors to rapidly generate strong T cell responses and thus the possibility of inducing potent CD8^+^ T cell responses was recently exploited in studies that utilized virus-Like Particles (VLP) loaded with three different recombinant proteins, KMP11 and LeishF3+, LJL143 from *Lutzomyia longipalpis* saliva in combination with an adjvant, GLA-SE, a TLR4 agonist (Cecílio et al., 2017). Results revealed that immunization with these VLP vaccines induced highly protective IFN-γ and TNF-α cytokines and subdued IL-10, IL-4 responses (Cecílio et al., 2017). Similarly, studies that included RNA encoding *Leishmania* antigens in addition to protein antigens in the immunization schedule generated MHCI-restricted T cell responses. Immunization with LEISH-F2-expressing RNA vaccine followed later by subunit vaccine afforded protection against challenge with *Leishmania donovani* (Duthie et al., 2018). Adeno viral vector mediated delivery of *Leishmania* antigens containing CD8 T cell epitopes has been explored based on the hypothesis that lack of appropriately targeted cell mediated immunity, including CD8+ T cell responses leads to the progression of VL and PKDL. An immunogencity study using adenoviral vectors expressing KMP-11 and HASP-B antigens showed strong induction of IFN-γ, TNF-α and IL-2 in an endemic cohort indicating that the viral vectors could be potent delivery agents as *Leishmania* vaccine antigens (Osman et al., 2017). Several of the anti-leishmanial vaccines evaluated the immunogenicity of the vaccines by measuring the induction of multifunctional CD4 and CD8 T cells based on the landmark report that described multifunctional Th1 responses were the best correlate of protection in experimental vaccines against cutaneous Leishmaniasis (Darrah et al., 2007). Accordingly, several of the aforementioned vaccine studies routinely measured multifunctional T cell responses as part of pre-clinical evaluation and in clinical studies. Vaccines that showed strong protection against needle challenge failed to protect when subjected to sandfly mediated challenge (Peters et al., 2009). This study highlighted the importance of incorporating sandfly challenge as part of evaluation of *Leishmania* vaccine efficacy which was not routinely performed in previous vaccine studies. Thus, the working group on research priorities for development of Leishmania vaccines put together by NIH/NIAID to deliberate on the target product profiles of *Leishmania* vaccines highlighted the role of sand fly vector in the vaccine development (Costa et al., 2011 **).**

**Figure 1:**
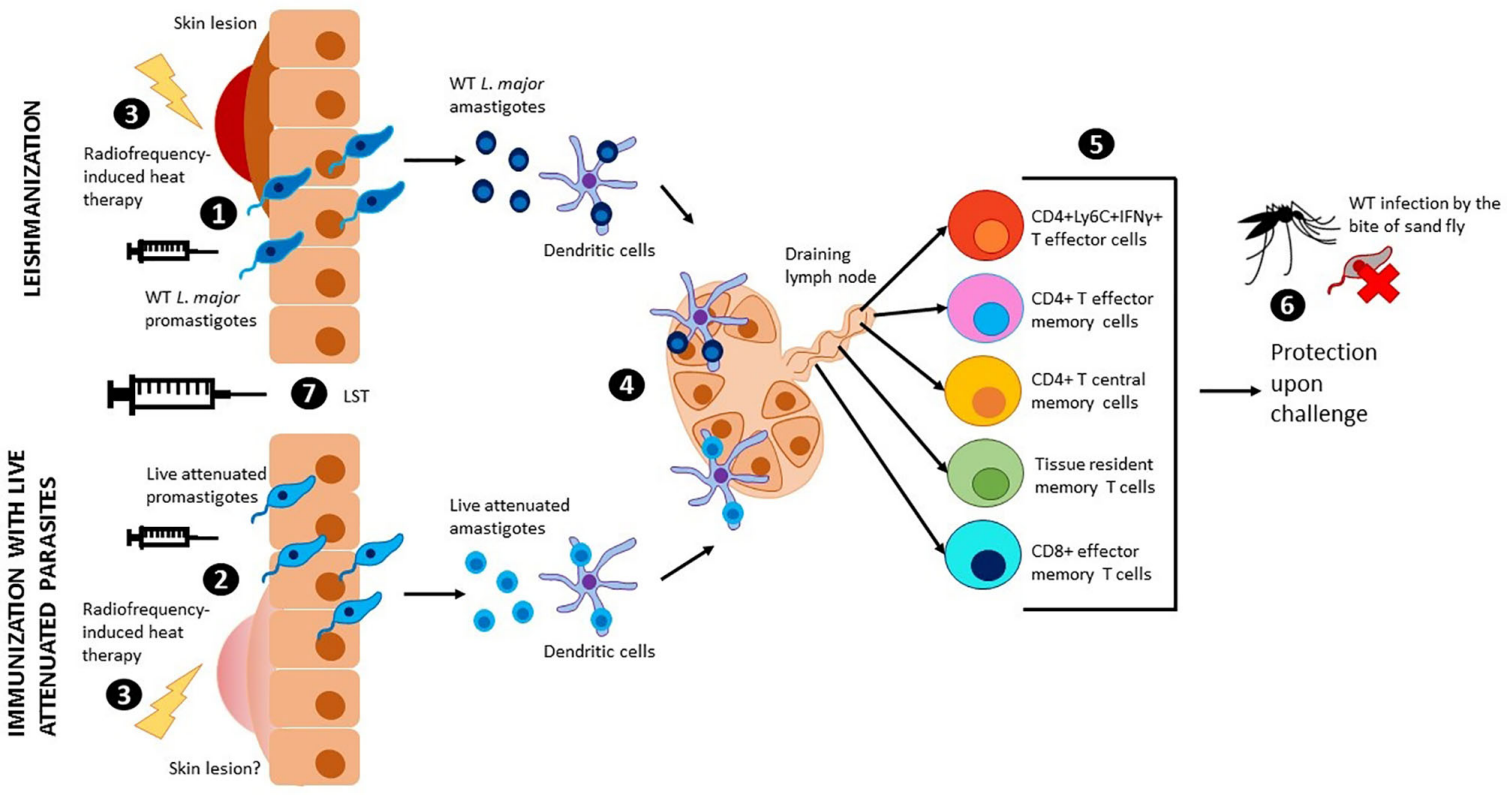
Leishmanization, immunization with live attenuated parasites and use of LST. Wild type *L. major* promastigotes (1) or live attenuted parasites (2) are injected intradermally through the skin. Wild type *L. major* parasites cause a skin lesion that can be controlled by radiofrequency-induced heat therapy (3). Injection with live attenuated parasites has shown no risk of skin lesions in pre-clinical models, however, radiofrequency-induced heat therapy can be used to mitigate this risk in clinical studies (3). Promastigotes transform into amastigotes and are internalized by dendritic cells, which travel to the draining lymph nodes to present the antigen to T cells (4). Different populations of effector and memory T cells are generated upon antigen presentation (5). Due to low parasitemia, these populations persist in the body and provide long term protection against sand fly challenge with virulent *leishmania* parasites (6). The leishmanin skin test (LST) can be used to evaluate cellular immunity and memory (7).

While the subunit vaccines against Leishmaniasis highlighted thus far were based on the highly successful vaccines against bacterial and viral agents, the empirical evidence with Leishmanization, deliberate inoculation with *Leishmania* parasites as a mean of acquiring protection against cutaneous leishmaniasis, overwhelmingly supports that a safer leshmanization method, if developed, would be an effective strategy (**Figure 1**). Earliest studies with inoculation of *Leishmania tropica* have shown that a lesión persisted for 3.5 to 13.5 months and upon follow up for two years, subjects that developed takes (scars developed at the site of immunization) showed strongest protection (80%) in a hyper endemic area of Iran (Nadim et al., 1983 **)**. Similarly, in a more recent study in Iran with inoculation of live *Leishmania major* showed that induced lesions persisted for up to 285 days with an induction of IFN-γ although in this limited cohort study such induction was highly variable (Khamesipour et al., 2005). Another study from Iran showed that leishmanization was found to reduce the incidence of the disease between one sixth and one eighth of its original level in a hyper-endemic region of Iran and thus was recommended for people at high risk of contacting the disease (Mohebali et al., 2019). Leishmanization was used in Israel, Iran, and Uzbekistan and showed to be effective to protect against future lesion development. A more detailed historical account of the ancient practice of Leishmanization is described in a previous review article (Mohebali et al, 2019). Results from these studies are corroborated by the experiments in resistant C57Bl/6 mice models that also enabled understanding of the immune mechanisms of protection. In studies with pre-clinical animal models, the immune response induced concomitant with the resolution of the cutaneous lesions has been shown to be most effective against sand fly mediated challenge (Peters et al., 2012). Further studies using this healed CL animal models revealed that the drivers of protection including the presence of effector CD4 T cell populations that secrete IFN-γ almost instantaneously following challenge infection, and that these Ly6C+CD4+ effector T cell populations are indefinitely maintained by the residual parasites following healing (**Figure 1**) (Peters et al., 2014; Hohman and Peters, 2019). In addition, parasite-independent memory T cells, including central memory T cells (T_CM_) and skin-resident T cells (T_RM_) have recently been described in leishmaniasis (**Figure 1**) (Colpitts and Scott, 2010; Glennie et al., 2015; Glennie and Scott, 2016; Glennie et al., 2017). Since the T_RM_ cells are skin resident, their isotropic distribution confers a distinct advantage in mediating protection due to their capacity to respond almost immediately upon challenge. Since maintaining a pool of effector T cells may not be feasible through vaccination such as recombinant vaccines that would not enable maintenance of persistent infection, strategies that boost the T_RM_ responses may be better achieved through vaccination (Scott, 2020). So far, the induction of T_RM_ cell populations was only shown in healed CL mice models indicating that Leishmanization, an analogous vaccination strategy may be similarly potent in inducing T_RM_ populations.

Despite the early promise, the practice of leishmanization was discontinued due to major complications including non-healing skin lesions, exacerbation of skin diseases, and the potential impact of immunosuppression (Seyed et al., 2018). The advent of more precise genetic manipulation methods in *Leishmania* enabled the development of genetically attenuated parasite strains that could be deployed as a surrogate for Leishmanization (**Figure 1**). Early iterations of the *Leishmania* gene deletion mutants showed promise in inducing strong protection against homologous and heterologous challenge (Silvestre et al., 2008). However, none of the genetically attenuated parasites were advanced to clinical trials due to unknown safety characteristics. Nevertheless, the potential of live attenuated *Leishmania* parasites vaccines is increasingly being recognized in recent analyses (Costa et al., 2011; Hohman and Peters, 2019). The ease of whole genome sequencing and the attendant capacity to monitor genome stability, phenotyping of attenuation of virulence, ability to propagate *Leishmania* parasites in bioreactors have significantly reduced the barriers towards large scale production and testing the genetically attenuated *Leishmania* parasites as candidate vaccines. Availability of FDA approved treatment methods such as radiofrequency-induced heat therapy have further provided tools for mitigating the risk if Leishmanization with live attenuated dermotropic *Leishmania* parasites were to result in unacceptable lesions (**Figure 1**) (Bumb et al., 2013). Our recent study showed that such genetically modified live attenuated *Leishmania* parasites with centrin gene deletion show comparable immune response as induced in a Leishmanization animal model (using virulent parasites) following a healed response, including the Ly6C^+^CD4^+^IFN-γ^+^ T cell (T effector cells) populations indicating that Leishmanization with such parasites could be a potent prophylactic while circumventing the safety issues associated with Leishmanization with live virulent parasites (**Figure 1**) (Zhang et al., 2020). Immunization with live *Leishmania* parasites has also shown potential with *L donovani* parasites (McCall et al., 2013; Karmakar et al., 2020). Inoculation with a dermotropic strain of *L donovani* isolated from Sri Lanka has shown that an immune response indicative of protection is induced, analogous to studies with *L major* parasites indicating a broader convergence of protective immune responses in both the species of parasites. To advance the live attenuated *L. major* parasites, further considerations of biomarkers of protection would be necessary. While the clinical studies with recombinant subunit vaccines provided an appropriate roadmap for clinical studies, a simpler method to test the immunogenicity would be helpful in rolling out live attenuated *Leishmania* parasite vaccines in clinical studies. Studies with vaccines against tuberculosis and DTH reaction against tuberculin reagent to monitor cell-mediated immunity provided a guide for developing similar method for evaluation of immunogenicity of *Leishmania* vaccines (Kaufmann, 2013).

## Alternative Methods For Determining The Efficacy of A L*eishmania* Vaccine

kAlthough there is a need for vaccination against both VL and CL, the priority should be VL since this is the fatal form of the disease. With respect to a vaccine for VL, India currently has among the highest number of new VL cases in the world (World Health Organization (WHO), 2020). Nevertheless, because of its dense population, the incidence rate in the highest endemic states such as Bihar is less that 1 case per 10,000 in the majority of endemic blocks. At this incidence rate, it would be impossible to perform a clinical phase 3 trial with a sufficient number of vaccinated and placebo individuals to determine the efficacy of any VL vaccine. With respect to CL, the countries with the highest numbers include Afghanistan, Brazil and Iran with an incidence of 8–10 times higher than VL. It may be more feasible to conduct an efficacy study in these countries if an appropriate site can be identified. It is nevertheless necessary to identify an alternative approach to determine whether a vaccine can generate a protective immune response. One approach may be to consider a controlled human infection model (CHIM) that could provide a pathway for accelerated vaccine development and to identify correlates of protection. Recently, *L. major* strains have been developed under GMP conditions and characterized for potential use in CHIM studies that can be used in CL vaccine trials (Ashwin et al., 2021). CHIM trials would not however be possible for VL due to the risk from challenge infections with a visceral disease causing *Leishmania* species such as *L. donovani.* It is reasonable to suggest however, that successful protection following vaccination in a *L. major* CHIM would provide evidence for immunity against VL (Zijlstra et al., 1994; Khalil et al., 2002). For VL, a potential approach is to use a surrogate marker of immunological protection such as the leishmanin skin test (LST).

## The Leishmanin Skin Test as a Marker for Cellular Immunity

The Leishmanin skin test (LST) involves a delayed-type hypersensitivity (DTH) skin reaction to antigen that is an indicator for cell-mediated immunity (**Figure 1**). Antigen induced DTH is used in epidemiologic investigations to determine exposure to pathogens such as *Mycobacterium tuberculosis* (*MTB*) and *Leishmania* (reviewed in (Vukmanovic-Stejic et al., 2006). The LST is similar to the tuberculin skin test (TST) and involves injection of antigen from *Leishmania* in the skin dermis resulting in a DTH reaction that is visually apparent.

The LST was then introduced in 1926 by Montenegro in the field of leishmaniasis as a diagnostic test for CL (Montenegro, 1926). During the DTH skin test, a small quantity of antigen is injected intradermally resulting in a local immune response that includes induration, swelling and inflammation within 24–72 h at the site of injection. The DTH is due to a Th1 type memory T cell response stimulating the release of IFN-γ, a potent stimulator of infiltrating macrophages resulting in the raised nodule/induration (Poulter et al., 1982; Cher and Mosmann, 1987).

It is noteworthy that in the case of a *Leishmania* exposure, the Th1 response in the skin occurs at the site of parasite inoculation by the sandfly. Like a latent *MTB* infection, a positive leishmanin skin test (LST) is concomitant with prior exposure to *Leishmania* and is positive after recovery from cutaneous leishmaniasis (CL) and visceral leishmaniasis (VL) (**Figure 1**). The ability to react positively to an LST lasts for many years following infection, unlike the antibody response that is transient lasting only several months. Notably, however, LST is negative in people with active VL disease indicating the absence of cell-mediated immunity against *L. donovani* and therefore the LST is not used as a diagnostic test for VL. Following successful treatment of VL, individuals convert to LST-positive and are immune for life. In the absence of symptomatic leishmaniasis, a positive LST indicates prior asymptomatic exposure to *Leishmania* and is strongly associated with immunological protection against developing visceral leishmaniasis in the future (see below). In the case of VL, most *L. donovani* infections remain asymptomatic and only about 10% of infections progress to symptomatic disease (Burza et al., 2018).

## Leishmanin Skin Test Positivity Following Natural Infection: Evidence for The Leishmanin Skin Test as a Surrogate Biomarker For Vaccine Efficacy

One study followed the migration of a cohort population from Western Sudan to Eastern Sudan a region endemic for *L. donovani* (Zijlstra et al., 1994). Most of the migrating population were LST-positive due to previous CL infections caused by *L. major* in Darfur. During a three-year follow-up, none of the LST-positive cohort (610 people) developed VL where 13 of the LST-negative cohort (172 people) did develop VL. Of the resident people in Eastern Sudan who had not migrated but were LST-positive due to successful treatment of VL (58 people), none developed VL compared to 17 new VL cases from 337 LST-negative people in the same cohort. Taken together, all 30 cases of VL (migrate or resident) were LST-negative and there were no cases in the LST-positive group. This provided strong evidence that LST-positivity resulting from *L. major* or *L. donovani* infection resulted in immunological protection against the development of VL caused by *L. donovani* in Sudan.

In a subsequent study performed in Eastern Sudan, during a 2-year longitudinal study comparing LST-positive and LST-negative cohorts, there were no cases in 109 LST-positive people compared to 55 cases out of 368 LST-negative people (Khalil et al., 2002). In a separate region, also in eastern Sudan during the same follow-up period, there were again no cases in a cohort of 192 LST-positive people compared to 7 cases in out of 529 LST-negative people. This corroborated the results from the earlier Zijlstra study in 1994 that people who have developed cellular immunity as demonstrated by a positive-LST were immune to developing VL in a highly endemic region of Eastern Sudan. Furthermore, the study showed that generation of LST-positivity due to an *L. major* infection provided protective immunity against VL caused by *L. donovani.* This is highly relevant, since it supports the notion that LmCen^-/-^ attenuated vaccine derived from *L. major* will provide protection against VL as discussed above in preclinical studies (Zijlstra, el-Hassan, Ismael and Ghalib 1994).

In Bangladesh, like in Sudan, VL is caused by infection with *L. donovani* and following successful treatment, people are generally immune for life and convert to LST-positive (Bern et al., 2006). People infected with *L. donovani* that do not develop symptoms (asymptomatic infections) are also LST-positive in the Indian subcontinent including Bangladesh, India, and Nepal. In 2002, of the 1,532 people tested for LST in the highly endemic district of Fulbaria, Bangladesh, 530 (35%) had a positive response of which 53 had been previously treated for VL (Bern et al., 2006). Only 1 of the 476 LST-positive cases developed VL during the following up compared to 43 from the 956 LST-negative developed VL. The observations from Bangladesh support the conclusion from the Sudan studies that a positive LST result confirms protective immunity against VL supporting that argument that the LST represents a biomarker for vaccine efficacy against VL.

Studies described above demonstrate that a positive LST is concomitant with pre-exposure to *Leishmania* and protection against VL. Currently however, the LST is no longer used in VL studies and *Leishmania* surveillance studies now involve detection of parasite DNA by molecular amplification methods or detection of antibodies using a DAT or the rK39 ELISA and rapid diagnostic test. These approaches, however, cannot effectively determine whether an individual has been previously exposed to the parasite since DNA may no longer be present following cure and the antibody response is transient. As the number of VL cases decline from the Indian subcontinent due to government elimination programs, it becomes increasingly difficult to measure efficacy in vaccine trials without a surrogate marker. Vaccine trial participants could therefore be subjected to the LST after vaccination to determine whether the vaccine has induced a protective immune response.

## Source of The Leishmanin Antigen for The Leishmanin Skin Test

Currently, there is no source of GMP grade leishmanin antigen available anywhere in the world and this is the major reason it is no longer used. One solution is that the same Biopharmaceutical facilities (Gennova Biopharmaceuticals, Pune, India) used to make GMP *LmCen* ^-/-^ vaccine discussed above can also be used to produce a GMP grade leishmanin antigen. Further, this leishmanin antigen can be tested in available animal models including vaccinated mice (Zhang et al., 2020) and hamsters (Karmakar et al., 2020). GMP grade leishmanin can also be tested on VL cured subjects (Karmakar et al., 2020) that provides a source of immune VL cases for validation prior to using it as a biomarker in vaccine studies. As facilities for culturing GMP grade live attenuated *Leishmania* vaccine is now advancing to phase 1 trial, this provides a unique opportunity to use the same facilities and regulatory processes to make a GMP grade leishmanin antigen for validation and widespread introduction into the field. Taken together, there is strong justification for re-introducing the LST into the field of leishmaniasis for surveillance and future vaccine trials.

In summary, leishmanization and the LST have been used successfully for decades but are no longer practiced. Newer technologies to make a safer second generation leishmanization vaccine and a more defined LST now justify the return of these interventions to support the elimination of leishmaniasis.

## Author Contributions

TP-F and GV co-authored the section on immune responses and composed the figure. SG co-authored the sections on immune response section, live attenuated vaccines, and reviewed the draft. PB co-authored the section on live attenuated vaccines and reviewed the draft versions. RD reviewed the draft versions. AS conceived the theme of the article and reviewed the draft versions. GM conceived the theme of the article, wrote the section on LST, and reviewed the draft versions. HLN conceived the theme of the article and reviewed the draft versions. All authors contributed to the article and approved the submitted version. All authors contributed to the article and approved the submitted version

## FundinG

This work is supported by NIH/NIAID grants R21 AI130485 02, RO3 AI144253 01 (AS), and Global Health Innovative Technology Fund grants G2018-201 and G2019-213 (AS, GM, HLN), and the Canadian Institutes of Health Research (CIHR153282) (GM).

## Disclaimer

Our contributions are an informal communication and represent our own best judgment. These comments do not bind or obligate FDA.

## Conflict of Interest

The authors declare that the research was conducted in the absence of any commercial or financial relationships that could be construed as a potential conflict of interest.
